# Literary Notes

**Published:** 1901-12

**Authors:** 


### LITERARY NOTES.

THE *American Journal of the Medical Sciences*, so long recognised as the leading monthly medical magazine in this country, has enlarged its space allotted to original communications, so that its November, 1901, issue contained double that usually set apart for the department referred to. The enlargement will be permanent. This magazine, always noted for its high-class original articles, abstracts, and reviews, will continue its energies in these directions, aiming in the future, as in the past, to stand in the front rank of medical periodicals.

---

W. B. SAUNDERS & Co., publishers, Philadelphia, announce the publication of Nothnagel's Encyclopedia of practical medicine, edited by Dr. Alfred Stengel, professor of clinical medicine in the University of Pennsylvania. The set will consist of ten or twelve volumes, translated by competent men who know English and German thoroughly, and who are acquainted with the subjects which they translate. Five or six volumes will appear during 1902 and the remainder soon afterward.

The usual method of publishers, when issuing a work of this kind, has been to compel physicians to take the entire system. This seems to us in many cases to be undesirable. Therefore, in purchasing this encyclopedia, physicians will be given the opportunity of subscribing for the entire system at one time; but any single volume or any number of volumes may be obtained by those who do not desire the complete series. This latter method, while not so profitable to the publisher, offers to the purchaser many advantages which will be appreciated by those who do not care to subscribe for the entire work at one time.

This American edition of Nothnagel's Encyclopedia will, without question, form one of the greatest systems of medicine ever produced, and the publishers feel confident that it will meet with general favor in the medical profession.

